# ERRγ-inducible FGF23 promotes alcoholic liver injury through enhancing CYP2E1 mediated hepatic oxidative stress

**DOI:** 10.1016/j.redox.2024.103107

**Published:** 2024-03-05

**Authors:** Yoon Seok Jung, Kamalakannan Radhakrishnan, Seddik Hammad, Sebastian Müller, Johannes Müller, Jung-Ran Noh, Jina kim, In-Kyu Lee, Sung Jin Cho, Don-Kyu Kim, Yong-Hoon Kim, Chul-Ho Lee, Steven Dooley, Hueng-Sik Choi

**Affiliations:** a Host-derived Antiviral Research Center, School of Biological Sciences and Technology, Chonnam National University, Gwangju 61186, Republic of Korea; b Molecular Hepatology Section, Medical Faculty Mannheim, Heidelberg University, Theodor-Kutzer-Ufer 1-3 (H42, Floor 4), 68167, Mannheim, Germany; c Department of Forensic Medicine and Veterinary Toxicology, Faculty of Veterinary Medicine, South Valley University, 83523 Qena, Egypt; d Center for Alcohol Research (CAR), University of Heidelberg, Im Neuenheimer Feld 350, 69120 Heidelberg, Germany; e Department of Functional Genomics, KRIBB School of Bioscience, Korea University of Science and Technology (UST), Daejeon 34113, Republic of Korea; f New Drug Development Center, Daegu-Gyeongbuk Medical Innovation Foundation, Daegu 41061, Republic of Korea; g Department of Internal Medicine, School of Medicine, Kyungpook National University, Kyungpook National University Hospital, Daegu 41944, Republic of Korea; h Research Institute of Aging and Metabolism, Kyungpook National University, Daegu 41940, Republic of Korea; i Center for Brain Disorders, Brain Science Institute, Korea Institute of Science and Technology (KIST), Seoul 02792, Republic of Korea; j Host-derived Antiviral Research Center, Department of Integrative Food, Bioscience and Biotechnology, Chonnam National University, Gwangju, 61186, Republic of Korea; k Laboratory Animal Resource Center, Korea Research Institute of Bioscience and Biotechnology, 125 Gwahak-ro, Yuseong-gu, Daejeon 34141, Republic of Korea

**Keywords:** ERRγ, Alcoholic liver disease, FGF23, CYP2E1, Oxidative stress

## Abstract

Fibroblast growth factor 23 (FGF23) is a member of endocrine FGF family, along with FGF15/19 and FGF21. Recent reports showed that under pathological conditions, liver produces FGF23, although the role of hepatic FGF23 remains nebulous. Here, we investigated the role of hepatic FGF23 in alcoholic liver disease (ALD) and delineated the underlying molecular mechanism. FGF23 expression was compared in livers from alcoholic hepatitis patients and healthy controls. The role of FGF23 was examined in hepatocyte-specific knock-out (LKO) mice of cannabinoid receptor type 1 (CB1R), estrogen related receptor γ (ERRγ), or FGF23. Animals were fed with an alcohol-containing liquid diet alone or in combination with ERRγ inverse agonist. FGF23 is mainly expressed in hepatocytes in the human liver, and it is upregulated in ALD patients. In mice, chronic alcohol feeding leads to liver damage and induced FGF23 in liver, but not in other organs. FGF23 is transcriptionally regulated by ERRγ in response to alcohol-mediated activation of the CB1R. Alcohol induced upregulation of hepatic FGF23 and plasma FGF23 levels is lost in ERRγ-LKO mice, and an inverse agonist mediated inhibition of ERRγ transactivation significantly improved alcoholic liver damage. Moreover, hepatic CYP2E1 induction in response to alcohol is FGF23 dependent. In line, FGF23-LKO mice display decreased hepatic CYP2E1 expression and improved ALD through reduced hepatocyte apoptosis and oxidative stress. We recognized CBIR-ERRγ-FGF23 axis in facilitating ALD pathology through hepatic CYP2E1 induction. Thus, we propose FGF23 as a potential therapeutic target to treat ALD.

## Abbreviations

Acox1: acyl-Coenzyme A oxidase 1
ADH: alcohol dehydrogenase
ALD: alcoholic liver disease
CB1R: cannabinoid receptor type 1
CCl_4_: carbon tetrachloride
CCl_4_-ALI: CCl_4_ mediated acute liver injury
cFGF23: C-terminal FGF23
CsA: cyclosporine A
CYP2E1: cytochrome P450 2e1
CYP4A10: cytochrome P450 4a10
ERRγ: Estrogen related receptor γ
ERRE: ERR response element
FA-AKI: folic acid induced acute kidney injury
FGF23: Fibroblast growth factor 23
FGFR: FGF receptor
iFGF23: intact FGF23
NOX2: NADPH oxidase 2
PTH: parathyroid hormone

## 1 Introduction

Alcohol related liver disease (ALD) is a global healthcare problem and encompasses a spectrum of economic, social as well as clinical consequences. ALD is caused by excessive drinking of the beverage alcohol (i.e., ethanol) for several years. Liver is the major site of alcohol metabolism through key ethanol-oxidizing enzymes alcohol dehydrogenase (ADH) and cytochrome P450 2e1 (CYP2E1) that exist in the cytosol and smooth endoplasmic reticulum, respectively [1,2]. The liver is highly susceptible to chronic alcoholic insult, which results in simple steatosis at early stage and concomitantly progresses to hepatitis, fibrosis, cirrhosis and more severe end stage liver diseases, including hepatocellular carcinoma [3]. Various pathways contributing to alcohol-mediated liver injury were identified till date, and alcohol-induced CYP2E1 is one of the central mechanisms instigating oxidative liver damage [4,5]. CYP2E1 exacerbates alcoholic liver disease through excessive ROS accumulation arbitrated oxidative damage and thereby triggers hepatic cell death [4,6,7]. The enzyme is highly expressed in the liver, especially in hepatocytes and Kupffer cells with high catalytic activity for metabolizing ethanol [8,9]. Inhibition of CYP2E1 protects the liver from alcohol-induced injury, as various CYP2E1 inhibitors, like polyenylphosphatidylcholine, chlormethiazole, diallyl sulfide and phenethyl isothiocyanate were hepatoprotective [6,[10], [11], [12], [13], [14], [15], [16]]. In contrast, overexpression of CYP2E1 exacerbates hepatic oxidative damage in alcohol exposed animals [17]. Expression of CYP2E1 is controlled by transcriptional, post-transcriptional, translational and post-translational regulation in response to various factors, including glucagon, insulin, leptin, adiponectin, growth factors, chronic diabetes and xenobiotics, like isoniazid, pyridine, acetone, pyrazole and ethanol [[18], [19], [20]]. In order to prevent or treat ALD, it is therefore, among others, important to identify safely druggable mediators of CYP2E1 induction, and gain knowledge about the detailed molecular mechanism how these are acting.

Estrogen related receptor γ (ERRγ), along with ERRα and ERRβ, belongs to the orphan nuclear receptor subfamily [21]. In general, ligand binding is mandatory for transcriptional activation of nuclear receptors. However, ERRγ displays constitutive activity without binding of a ligand, due to a permanently activated conformation of its ligand-binding domain [22]. ERRγ exerts its biological function in many vital organs, including liver, skeletal muscle, heart, pancreas, brown adipose tissue and brain [23]. ERRγ regulates hepatic fibroblast growth factor 23 (FGF23), an endocrine hormone primarily secreted by osteocytes and osteoblasts, but also induced in hepatocytes in response to folic acid induced acute kidney injury (FA-AKI) and carbon tetrachloride (CCl_4_) mediated acute liver injury (CCl_4_-ALI) [24,25]. Hepatocyte specific ablation of ERRγ or inverse agonist mediated inhibition of ERRγ transactivation significantly reduced hepatic FGF23 production and plasma FGF23 levels in both FA-AKI and CCl_4_-ALI mice. Various cellular signals, like alcohol, inflammation, fasting and feeding may activate ERRγ via membrane located cannabinoid-, IL-6-, glucagon- and insulin-receptors [23]. We previously showed in different reports that alcohol induces ERRγ expression through cannabinoid receptor type 1 (CB1R) [26,27] and that ERRγ regulates FGF23 expression in the liver [24,25]. It will be interesting to know if there exists a CBIR-ERRγ-FGF23 axis in alcohol-induced liver injury.

FGF23 is primarily produced from bone and regulates phosphate homeostasis and vitamin D metabolism through its action on kidney [28]. The biologically active form of FGF23, intact FGF23 (iFGF23), is a 30–32 kDa protein, which is cleaved by protein convertases between arginine 179 and serine 180 into inactive 18 kDa N-terminal and 12 kDa C-terminal FGF23 (cFGF23) [29]. FGF23 primarily acts on proximal tubular cells of the kidney via the FGF receptor (FGFR) and its co-receptor α-klotho. FGF23 signaling facilitates phosphate excretion through downregulating renal expression of sodium phosphate co-transporters NaPi-2a and NaPi-2c in brush border member of the proximal tubule. Parathyroid hormone (PTH) and vitamin D induce FGF23 expression in bone, whereas in return FGF23 inhibits vitamin D and PTH production, both in kidney and parathyroid gland. PTH enhances vitamin D biosynthesis, whereas conversely, vitamin D inhibits PTH production. This indicates that a number of negative feedback loops exist among FGF23, PTH and vitamin D [30]. Systemic levels of FGF23 are massively elevated in kidney disease conditions and positively correlate with deleterious clinical outcomes, including disease progression and mortality [31]. FGF23 is also produced by non-osseous tissues, including pathological conditions of kidney and liver, e.g. FA-AKI, CCl_4_-ALI, autosomal dominant polycystic kidney disease and childhood biliary atresia [24,25,[32], [33], [34], [35]]. Circulatory FGF23 levels are increased among alcoholics [36,37], however the source, mechanism of regulation and role of FGF23 in ALD remains to be delineated.

In this study, we show that ERRγ mediated hepatic FGF23 accelerates liver injury in response to chronic alcohol feeding in mice. Additionally, through utilizing three diverse hepatocyte-specific knock-out (LKO) mice, CB1R-LKO, ERRγ-LKO and FGF23-LKO, we revealed existence and importance of a CB1R-ERRγ-FGF23 signal transduction axis in alcoholic liver injury. Overall, our observations suggest that FGF23 may represent a novel therapeutic target for the treatment of ALD.

## 2 Materials and methods

### 2.1 Materials

GSK5182 was synthesized as described previously [38] and used at a concentration of 40 mg/kg for *in vivo* or 10 μM for *in vitro* experiments. Cyclosporine A (CsA) (Sigma-Aldrich Chemical Co., St Louis, MO, USA) used at a concentration of 1 μg/ml for *in vitro* experiments. Arachidonyl-2′-chloroethylamide (ACEA) was purchased from Tocris Bioscience and used at a concentration of 10 μM for *in vitro* experiments.

### 2.2 Ethics approval

The patients study protocol was approved by the university of Heidelberg under the title Nichtinvasive Leberfettbestimmung mittels Controlled Attenuation Parameter with approval number S-150/2015. All animal procedures were approved by the Institutional Animal Care and Use Committee of Korea Research Institute of Biosciences and Biotechnology (KRIBB, KRIBB-AEC-22175). All animal experiments were performed in accordance with the Guide for the Care and Use of Laboratory Animals published by the US National Institutes of Health.

### 2.3 Alcoholic hepatitis patients

All patients were Caucasian heavy drinkers (>80 g per day in males and >60 g per day in females) with a mean alcohol consumption of 183 g/day and presented for alcohol detoxification therapy from 2007 until 2020 at Salem Medical Center Heidelberg. The study protocol was reviewed and approved by the local Ethics Committee (S434-2006) and S150-15) and all patients gave written informed consent prior to inclusion. In all patients, routine laboratory markers, abdominal ultrasound and liver stiffness (Fibroscan, Echosens SA, Paris, France) were studied over a mean detoxification period of 6.9 ± 2.0 days. From 40 patients enrolled between 2014 and 2020. In 25 additional patients, enrolled between 2007 and 2010, liver biopsy was performed according to the percutaneous Menghini technique under ultrasound guidance. For histological analysis, the specimen was fixed in formalin and embedded in paraffin. Additionally, scoring of steatosis, inflammation, hepatocellular ballooning, and fibrosis stage was performed as described by Kleiner et al. [39].

### 2.4 Gene Expression Omnibus (GEO) datasets analysis

The gene expression values of estrogen related receptor gamma (ERRγ), fibroblast growth factor 23 (FGF23), cytochrome P450 family 2 subfamily E member 1 (CYP2E1), brevican (BCAN), SPARC like 1 (SPARCL1), aldehyde dehydrogenase 2 family member (ALDH2) and alcohol dehydrogenase 1B (class 1), beta polypeptide (ADH1B) were obtained from publicly available Gene Expression Omnibus (GEO) database from National Center for Biotechnology Information (NCBI). Two gene expression datasets GSE143318 and GSE167308 were analyzed for alcoholic hepatitis and alcoholic cirrhosis patients respectively [40,41]. Obtained gene expression values were represented as violin plots using GraphPad Prism8 software.

### 2.5 Animal studies

Eight-week-old male mice were used for all experiments. C57BL/6J wild-type (WT) mice were obtained from KRIBB (KRIBB, Daejeon, Korea). C57BL/6J mice containing floxed CNR1 (CNR1 f/f) mice were generated as described previously [42]. C57BL/6J mice containing floxed ERRγ exon 2 (ERRγ f/f) were obtained from PHENOMIN-iCS, PHENOMIN, the French National Infrastructure in Biology and Health (Illkirch, France). C57BL/6J mice containing floxed FGF23 exon 2 (FGF23 f/f) were obtained from Jackson Laboratories (Bar Harbor, ME, USA). To generate the hepatocyte-specific CNR1 knockout (CB1R-LKO), the hepatocyte-specific ERRγ knockout (ERRγLKO), or the hepatocyte-specific FGF23 knockout (FGF23-LKO) mice, CNR1 f/f, ERRγ f/f, or FGF23 f/f animals were crossbred with C57BL/6J-Alb-Cre transgenic mice, which express Cre recombinase in hepatocytes under the control of the albumin promoter (Jackson Laboratories). Prior to the experiments, mice were acclimatized to a 12-h light/dark cycle at 22 ± 2 °C for 2 weeks with unlimited food and water in a specific pathogen-free facility. For chronic alcoholic-induced liver injury model, WT, CB1R-LKO, ERRγ-LKO, or FGF23-LKO mice were pair-fed an alcohol-containing Lieber-DeCarli formulation-based liquid (Dyets, Bethlehem, PA, USA) diet or control liquid diet (Dyets) for 6 weeks with a stepwise feeding procedure. Mice were fed liquid diet with gradually increased alcohol concentration in the first 2 weeks to reach 4% (w/v) alcohol and were continually on the diet for 4 weeks. For GSK5182 experiments, during the 6 weeks of feeding with alcohol (4%, w/v) liquid diet, GSK5182 (40 mg/kg in 30% PEG400, p.o., once-daily) was injected for the last 2 weeks into mice. For recombinant human FGF23 (rhFGF23) experiment, WT mice were injected intravenously with rhFGF23 (40 μg/kg in PBS; R&D system; 2604-FG/CF), then animals were sacrificed at 0.5, 1, 3, 6, 12, or 24 h after rhFGF23 treatment.

### 2.6 FGF23 RNAScope and image analysis

In situ detection of FGF23 mRNA transcription was done with the RNAscope Multiplex Fluorescent Detection Kit v2 (ACD/Bio-Techne; 323100) according to the manufacturer's protocol with some modifications. Briefly, formalin-fixed paraffin-embedded sections from human livers were cut by a microtome. Then, the slides were baked in an incubator at 60 °C for 1h. After deparaffinization with xylene and rehydration with ethanol, the slides were incubated for 5 min at 60 °C. Subsequently, 5–8 drops of H_2_O_2_ were added, such as to cover each section, and incubated for 10 min. After the washing steps, 5 drops of Protease Plus were added, such as to cover each section, and incubated for 30 min at 95 °C in an oven. Hybridization was initiated by adding the RNA probes (MmHs-FGF23, ACD/Bio-Techne; 557241, probes C1, C2, and C3) on the sections. Incubation was performed at 40 °C in an oven for 2 h. After the washing steps, the slides were successively covered with 4–6 drops of Amp1, Amp2 and Amp3 each, and incubated at 40 °C in an oven for 30 min, 30 min and 15 min, respectively, with each 2 washing steps in between. Then the slides were successively incubated with HRP-C1 and Opal 520 fluorophore (Akoya Biosciences, FP1487001KT) for 15 min and 30 min, respectively. Subsequently, a few drops of HRP blockers were added to the slides and incubated for 5 min. Then the slides were counterstained with DAPI and mounted with prolong gold anti-fade mounting media. An Aperio 8 Slide scanner (Leica) was used for image documentation and quantification. 6 images from FGF23 expressing regions from each of 5 liver samples from healthy controls and 7 from patients with alcoholic related liver disease were quantified using image J. Data are presented as a percentage of positive FGF23 pixels from the total captured area.

### 2.7 Recombinant adenoviruses

Ad-US were described previously [43]. In brief, the cDNA encoding ERRγ was cloned into a pAd-YC2 shuttle vector contains a bovine growth hormone polyadenylation signal sequence under the control of the cytomegalovirus (CMV) promoter. Adenovirus short hairpin ERRγ (Ad-shERRγ) was generated with pAd-easy system. The target sequence of shERRγ is GAACGGACTGGACTCGCCACCTCTCTA [44]. All virus constructs were purified by cesium chloride density gradient combined with ultracentrifugation. Ad-GFP and Ad-FLAG-ERRγ were infected in cells (1 × 10^8^ PFU) and mice (3 × 10^9^ PFU/mouse). For ERRγ knockdown experiments, cells were infected with Ad-US and Ad-shERRγ (1 × 10^8^ PFU) for 48 before the treatment protocol.

### 2.8 Cell culture and transient transfection assays

Huh7 (human hepatoma cells) and AML12 (mouse immortalized hepatocytes) cells were obtained as described previously [45]. The cells were maintained in a humidified atmosphere containing 5% CO_2_ at 37 °C and used for experiments at 75% confluence. Transient transfections were performed using Lipofectamine 2000 (Invitrogen, Carlsbad, CA, USA) according to the manufacturer's instructions. The cells were treated with 10 μM GSK5182 unless noted otherwise. After 48 h of transfection, the cells were harvested, and luciferase activity was measured and normalized to β-galactosidase activity. All cell culture experiments were performed as three independent biological replicates.

### 2.9 RNA isolation and analysis

Total RNA was isolated using the TRIzol reagent (Invitrogen, CA, USA) according to the manufacturer's instructions, and used as template to synthesize cDNA with TOPscript RT DryMix (dT18 plus; Enzynomics, Daejeon, Korea). Reverse transcription was performed at 37 °C for 5 min, 45 °C for 60 min and 95 °C for 5 min on a thermocycler (TaKaRa, Shiga, Japan). The cDNAs were analyzed using the Applied Biosystems StepOnePlus real-time PCR system (Applied Biosystems, Foster City, CA, USA) and Power SYBR Green PCR Master Mix (Applied Biosystems). Based on the obtained CT values, the mRNA expression was calculated using the 2^−ΔΔ^CT method. Sequences of primers used in this study were listed in the Supplementary Table 1.

### 2.10 Western blot analysis

Mouse tissues were lysed with using RIPA buffer (Elpis-Biotech, Daejeon, Korea) and subjected to immunoblot analysis as described previously [46]. Proteins from liver whole and cytosolic fraction lysates were separated by 10% SDS- PAGE and transferred to nitrocellulose membranes. The membranes were probed with anti-β-actin (AbFrontier; diluted 1:5000) [26], anti-CYP2E1 (Proteintech, 19937-1-AP, 1:1000), anti-Cleaved caspase3 (Cell signaling, #9661; diluted 1:1000), anti-Cytochrome C (Cell signaling, #11940; diluted 1:1000), anti-smac (Abcam, ab32023; diluted 1:1000), and anti-α-tublin (AbFrontier; diluted 1:5000). The membranes were probed with specified antibody. Immunoreactive proteins were visualized using an Amersham ECL kit (GE Healthcare, Piscataway, NJ, USA) or using iBright CL1000 imaging system (Invitrogen) according to the manufacturer's instructions.

### 2.11 Immunohistochemical analysis

Mouse liver samples were fixed in 10% neutral buffered formalin, embedded in paraffin, cut into 5 μm-thick sections. To detect ERRγ and FGF23 protein, liver sections were stained with an anti-ERRγ and anti-FGF23 antibody (R&D Systems, Minneapolis, MN, USA) and visualized using 3,3′-diaminobenzidine. Images were captured using a light microscope (BX51; Olympus Corporation). Immunofluorescence staining for 4-hydroxynonenal (4-HNE) was carried out using anti-4-HNE antibody (Abcam, Cambridge, MA, USA) and visualized with AlexaFluor 488-nm antibody (Invitrogen, Carlsbad, CA, USA). The samples were analyzed by confocal microscopy (LSM 800; Carl Zeiss, Jena, Germany), and the intensity of green fluorescence was measured and averaged in 2 randomly selected field at ×200 magnification by an image analysis program (Image Inside; GS media, Daejeon, Korea).

### 2.12 Chromatin immunoprecipitation assay

SimpleChIP Plus Enzymatic Chromatin IP kit (catalog # 25268S) was used to carry out chromatin immunoprecipitation (ChIP) analysis, as previously described [47], in vehicle and ethanol treated mice liver tissues. Real-time qPCR analysis of ChIP eluded and input DNA was performed using the following primers. Mouse FGF23 ERRE site (−512 to −327), forward 5′-AAACAAGGACACTGGAGGGAGATG-3′ and reverse, 5′-CCTCAATTTCAAGCCAGTGCTCC-3’.

### 2.13 In vivo imaging

WT mice were infected with adenovirus fused with mouse FGF23 promoter WT-luciferase (Ad-FGF23 WT-luc) or mouse FGF23 promoter ERRE mutant-luciferase (Ad-FGF23-ERREmut-luc) via tail vein injection (2.5 × 10^9^ PFU/mouse). Four days of post-injection, mice either received a single dose of ethanol (6 g/kg) or vehicle intragastrically to induce alcohol-associated liver injury. Mice were imaged using an IVIS Lumina III imaging system (Caliper Life Sciences, Hopkinton, MA, USA) as described previously [47].

### 2.14 Caspase-3 activity

Caspase-3 activity was assessed using a colorimetric assay kit (Abcam, ab39401, Cambridge, UK) according to the manufacturer's instructions. Briefly, liver tissues from vehicle or ethanol treated WT and FGF23-LKO mice were homogenized and added into the 96-well plate in duplicates. Into each well, 50 μL of 2× reaction mixture containing DTT and 5 μL of chromophore *p*-nitroaniline (p-NA) labeled substrate DEVD-pNA were added, followed by incubation at 37 °C for 120 min 2× reaction buffer only was added into the control wells. All wells were measured on a microplate reader at 405 nm absorbance. Data are presented as fold change with respect to the WT-control group.

### 2.15 Terminal transferase dUTP nick end labeling (TUNEL) assay

TUNEL assay was performed using sections of paraffin-embedded tissue samples according to the method of ApopTag plus peroxidase in situ apoptosis detection kit (Chemicon International, Temecula, CA, USA). For detection of apoptotic cells, fragmented DNA of apoptotic cells were deoxygenated by terminal deoxynucleotidyl transferase (TdT). The digoxigenin was labeled by anti-digoxigenin-peroxidase, and visualized by DAB. Images were captured using a light microscope (BX51; Olympus Corporation). TUNEL-positive hepatocytes were counted and averaged in at least 20 randomly selected fields at ×200 magnification.

### 2.16 Blood analysis

Levels of plasma alanine aminotransferase (ALT), aspartate transaminase (AST), blood urea nitrogen (BUN) and creatinine were determined using an automated blood chemistry analyzer (AU480; Beckman Coulter, Krefeld, Germany). Levels of plasma phosphate levels were determined via ELISA kit, according to the manufacturer's protocol (DIPI-500, BioAssay Systems, CA, USA). Plasma-intact FGF23 levels were determined via ELISA, according to the manufacturer's protocol (60–6800, San Diego, CA, USA).

### 2.17 Isolation of cytosolic fractions

Liver tissues were homogenized with hypotonic buffer (0.5 M HEPES, 1 M MgCl2, 1 M KCl, 1 M DTT and dH2O) containing the protease inhibitor. The tissue homogenates were centrifuged at 700×*g* for 10 min at 4 °C to pellet the nuclei. The supernatant of homogenate was centrifuged at 5000×*g* for 10 min at 4 °C to precipitate the mitochondria. The supernatant which containing cytosolic fraction was kept at −80 °C for further experiments.

### 2.18 Determination of total ROS production

The total levels of ROS were measured via the oxidative conversion of the nonfluorescent 2′, 7′-dichloro-dihydro-fluorescein diacetate (DCFHDA; Invitrogen, Carlsbad, CA, USA) to the highly fluorescent 2′, 7′-dichlorofluorescein in the cytosolic liver fractions. Liver extracts were incubated at 37 °C for 30 min with a 5 μM DCFHDA. Subsequently, fluorescence intensity was measured at 485 nm excitation and 530 nm emission using a SpectraMax iD5 Multi-Mode Microplate Reader (Molecular Devices, San Jose, CA, USA) and normalized to the protein content.

### 2.19 Statistical analyses

Data were analyzed with Prism 8 (GraphPad Software, La Jolla, CA, USA) and presented as mean ± SD. Comparison between two groups was performed using the two-tailed Student's t-test, whereas comparison between multiple groups was conducted via ordinary one-way ANOVA with Tukey's multiple comparison test. Differences were considered statistically significant at p < 0.05.

## 3 Results

### 3.1 Hepatic FGF23 expression is increased in alcoholic liver disease

**Fig. 1:**
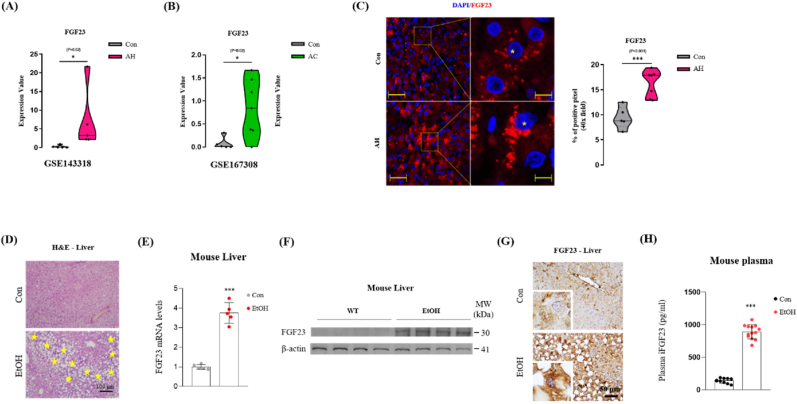
Hepatic FGF23 gene expression increased in alcoholic liver injury. (A, B) Hepatic levels of FGF23 expression in alcoholic hepatitis and cirrhosis patients compared to healthy controls using two datasets (GSE143318-alcoholic hepatitis; GSE167308-alcoholic cirrhosis) obtained from the Gene Expression Omnibus (GEO) database. (C) Representative image and quantification of FGF23 RNAscope fluorescent in situ hybridization of liver sections from healthy persons (con; n = 5) and alcoholic hepatitis patients (AH; n = 7). Red dots represent FGF23 RNA molecules. DAPI (blue) was added to label cell nuclei. Scale bar is 50 μm. Scale bar of the inlay is 10 μm. The yellow asterisks refer to hepatocyte nuclei. FGF23 pixels were quantified using image J, as described in M + M. (D–H) WT mice were treated with vehicle or ethanol. (D) Representative image of hematoxylin and eosin (H&E) staining in liver sections. Asterisk indicates vacuolar degeneration of hepatocytes. (E) Quantitative PCR analysis of FGF23 mRNA level in total RNA isolated from mouse livers (n = 5 per group). (F) Western blot analysis of FGF23 protein levels in liver tissues (n = 4 per group). (G) Representative images of FGF23 immunohistochemical analysis in liver sections. (H) Plasma iFGF23 level was measured by ELISA (Control n = 10; EtOH n = 13). The data were expressed as the mean ± SEM and analyzed using two-tailed Student's *t*-test. *p < 0.05; **p < 0.01; ***p < 0.001.

The levels of FGF23 in circulation have been reported to be higher in alcoholic patients compared to healthy controls [36,37]. However, the source and mechanism of alcohol-induced FGF23 production and its role in ALD remain largely unknown. In our previous studies, we reported that ERRγ expression is upregulated in alcoholic liver disease [26], and ERRγ is a transcriptional regulator of hepatic FGF23 production in mice with FA-AKI and CCl_4_-ALI [25,47]. Based on these findings, we hypothesized that ERRγ may regulate hepatic FGF23 production in response to alcohol feeding. To test this hypothesis, first we examined the expression level of FGF23 in publicly available Gene Expression Omnibus (GEO) datasets of alcoholic hepatitis (GSE143318) and alcoholic cirrhosis (GSE167308) patients [40,41]. Our analysis revealed that the expression values of FGF23 was higher in the livers of ALD patients compared to controls (Fig. 1A and B). As a validation control, we also assessed the expression levels of two genes each that were previously reported as up-regulated (BCAN and SPARCL1) and down-regulated (ALDH2 and ADH1B) in stated conditions (Figs. S1A and B). Next, we measured FGF23 mRNA level in ALD patients using RNAscope fluorescent in situ hybridization. We found that, in the liver, FGF23 is mainly produced in hepatocytes and significantly upregulated in ALD patients compared to healthy controls (Fig. 1C). To confirm the effect of alcohol on hepatic FGF23 and to determine the role of alcohol-induced FGF23, we generated a chronic alcohol-induced liver injury model by feeding the mice with liquid diet, gradually increasing the alcohol concentration in the first 2 weeks to reach 4% (w/v) alcohol and then continued for 4 weeks. H&E staining of mouse liver and kidney sections shows that chronic alcohol feeding induced liver injury with macrovesicular steatosis, accompanied by ballooning degeneration of hepatocytes (Fig. 1D; S1C and D) without causing any obvious damage to kidney (Figs. S1E–G). ln harmony with human data, FGF23 mRNA and protein expressions were found to be upregulated in the livers of ALD mice compared to control (Fig. 1E–G). Moreover, plasma FGF23 level was found to be upregulated as the result of hepatic FGF23 gene upregulation (Fig. 1H). To investigate potential zonation differences in FGF23 production by the liver, we initially examined basal FGF23 expression levels across various zones of human liver tissues using the dataset GSE105127 obtained from the publicly available Gene Expression Omnibus (GEO) database. Notably, basal FGF23 exhibited high expression in the periportal zone, and a weaker expression in intermediate and pericentral zones (Fig. S2A). Subsequently, we evaluated FGF23 expression levels in distinct zones of livers from mice treated with either vehicle or ethanol through immunohistochemistry. In the control group, FGF23 expression was similar across the three zones of the hepatic lobule, whereas chronic alcohol feeding induced FGF23 expression at highest levels in the periportal area (Fig. S2B). Intriguingly, the expression pattern of alcohol-induced FGF23 production in mouse livers mirrored the basal FGF23 expression pattern observed in human livers. Overall, our findings suggest that the hepatic expression of FGF23 was upregulated in response to alcoholic liver injury.

### 3.2 Activated CB1R-induced ERRγ transcriptionally regulates hepatic FGF23 in response to alcohol

**Fig. 2:**
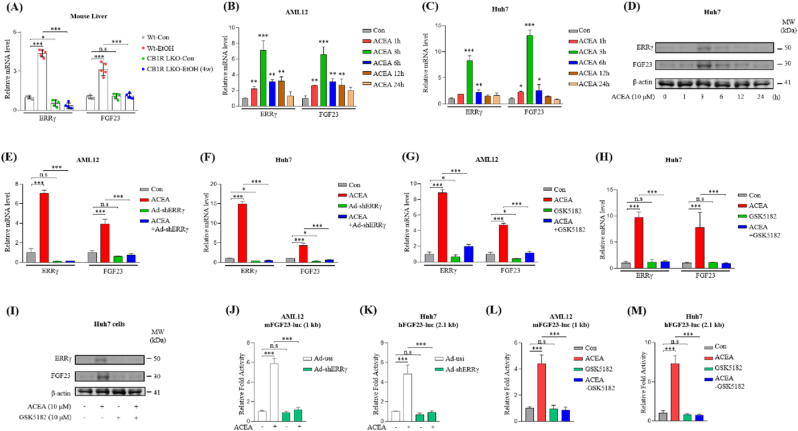
Activation of the CB1 receptor induces FGF23 gene expression through ERRγ in hepatocytes. (A) Quantitative PCR analysis of total RNA isolated from control or ethanol fed wild type and CBIR-LKO mice (n = 5 per group). (B and C) Quantitative PCR analysis of total RNA isolated from (B) AML12 and (C) Huh7 cells treated with arachidonyl-2′-chloroethylamide (ACEA) (10 μM) in time dependent manner. (D) Western blot analysis of ERRγ and FGF23 protein levels in Huh7 cells treated with ACEA (10 μM) in time dependent manner. (E and F) Quantitative PCR analysis of total RNA isolated from (E) AML12 and (F) Huh7 cells, infected with Ad-US or Ad-shERRγ for 36 h, and then treated with ACEA for 3 h. (G and H) Quantitative PCR analysis of total RNA isolated from (G) AML12 and (H) Huh7 cells treated with ACEA in presence or absence of GSK5182. (I) Western blot analysis of ERRγ and FGF23 protein levels in Huh7 cells treated with ACEA in presence or absence of GSK5182. (J and K) Ad-Usi or Ad-shERRγ infected (J) AML12 and (K) Huh7 cells were transfected with mFGF23-luc or hFGF23-luc respectively, and then stimulated with ACEA for 3 h. (L) AML12 and (M) Huh7 cells were transfected with the mFGF23-luc or hFGF23-luc respectively, and stimulated with ACEA for 3 h, in presence or absence of GSK5182. All *in vitro* experiments were conducted in triplicates as independent biological replicates. The data were expressed as the mean ± SEM and analyzed using two-tailed Student's *t*-test (B and C) or ordinary one-way ANOVA with Tukey's multiple comparisons test (A and D-K). *p < 0.05; **p < 0.01; ***p < 0.001; not significant (n.s).

We previously showed that activation of hepatic CB1R signaling induces ERRγ gene expression in chronic alcohol fed mice [26]. To confirm this CB1R-ERRγ signaling axis and its organ specificity in our current model of ALD, we measured ERRγ mRNA expression in major tissues including brain, heart, lung, liver, spleen, kidney, bone and muscle of ALD mice. Interestingly, similar to FGF23, the expression of ERRγ mRNA also specifically induced in the liver, while not in other major organs of ALD mice (Fig. S3A). Moreover, we validated the ERRγ gene expression in the GEO datasets of alcoholic hepatitis (GSE143318) and alcoholic cirrhosis (GSE167308) patients, which we used to check FGF23 expression. Expression of ERRγ was found to be higher in the livers of ALD patients compared to controls (Figs. S3B and S3C). Interestingly, similar to ERRγ, the expression of FGF23 mRNA also specifically induced in the liver, while not in other major organs of ALD mice (Fig. S3D). Based on these observations, we hypothesized that activated CB1R-induced ERRγ expression may regulate hepatic FGF23 gene expression in response to alcohol. To test our hypothesis, WT and CB1R-LKO mice were treated with ethanol for 4 weeks and we measured ERRγ and FGF23 mRNA expression in liver tissue. Ethanol treatment significantly induces ERRγ and FGF23 mRNA expression in WT mice, however, loss of CB1R expression abolished the ethanol effect on ERRγ and FGF23 expression (Fig. 2A). Next, we treated immortalized mouse hepatocytes (AML12) and human hepatoma cells (Huh7) with CB1R agonist ACEA and tested ERRγ and FGF23 gene expression in a time-dependent manner. Both are induced with maximal expression after 6 h of ACEA (10 μM) treatment in AML12 and Huh7 cells (Fig. 2 B and C). Moreover, ERRγ and FGF23 protein levels were also induced in time dependent manner in Huh7 cells in response to ACEA treatment (Fig. 2D). Knockdown of ERRγ expression using Ad-shERRγ or inhibition of ERRγ transactivation using ERRγ-specific inverse agonist (GSK5182) inhibited ACEA induced up-regulation of ERRγ and FGF23 gene expression (Fig. 2E–H). ACEA induced ERRγ and FGF23 protein levels were found to be inhibited in presence of GSK5182 in Huh7 cells (Fig. 2I). To expose the molecular mechanism of ERRγ mediated FGF23 gene expression, we cloned the mouse and human FGF23 gene promoter into luciferase reporter plasmids and transfected AML12 and Huh7 cells respectively. FGF23 promoter activity is significantly induced by ACEA treatment, however Ad-shERRγ or GSK5182 treatment completely abolished ACEA-induced FGF23 promoter activity (Fig. 2J–M). Taken together, our results indicate that activated CB1R-induced ERRγ transcriptionally upregulates hepatic FGF23 gene expression.

### 3.3 ERRγ is required for alcohol mediated up-regulation of hepatic FGF23 expression

**Fig. 3:**
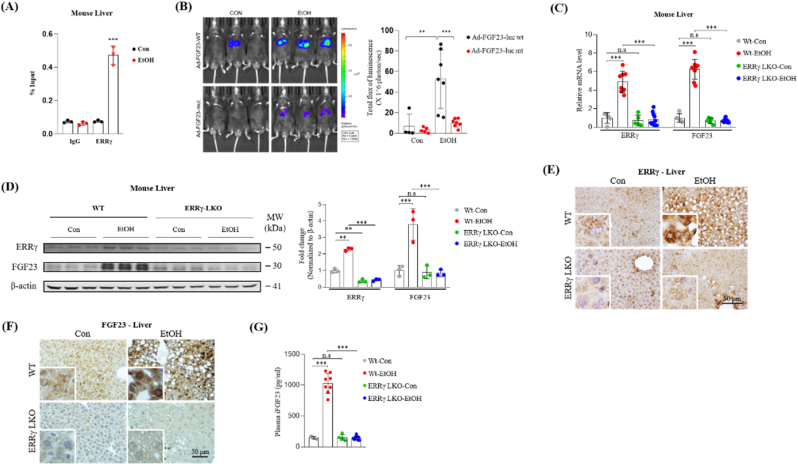
Chronic alcohol feeding-induced FGF23 is inhibited by hepatocyte specific ERRγ deficiency. (A) *In vivo* chromatin immunoprecipitation (ChIP) assay showing the binding of ERRγ to FGF2 gene promoter in response to ethanol. WT mice were treated with control or ethanol, and soluble chromatin was immunoprecipitated with ERRγ antibody followed by qPCR with primers corresponding to ERRE region of mouse FGF23 gene promoter. (B) Representative images for *in vivo* imaging of hepatic FGF23 promoter WT-luciferase (Ad-FGF23 WT-luc) and ERRE mutant-luciferase (Ad-FGF23 mut-luc) activity in control or ethanol treated mice. (C–F) WT and hepatocyte-specific ERRγ knock-out mice (ERRγ-LKO) were exposed to vehicle or alcohol and sacrificed for analysis (Wt-Con n = 5, Wt-EtOH n = 9, ERRγ-LKO-Con n = 5, ERRγ-LKO-EtOH n = 9). (C) Quantitative PCR analysis of ERRγ and FGF23 mRNA levels in total RNA isolated from mouse livers. (D) Western blot analysis and quantification of ERRγ and FGF23 protein levels in liver tissues. (E and F) Representative images of (E) ERRγ and (F) FGF23 immunohistochemical analysis in liver sections. (G) Plasma iFGF23 levels were measured by ELISA. The data were expressed as the mean ± SEM and analyzed using ordinary one-way ANOVA with Tukey's multiple comparisons test. **p < 0.01; ***p < 0.001; not significant (n.s).

As previous *in vitro* results revealed that alcohol-induced FGF23 promoter activity was hampered by Ad-shERRγ or GSK5182 treatment (Fig. 2J–M), we investigated the *in vivo* binding of ERRγ to the FGF23 gene promoter in response to alcohol. Chromatin immunoprecipitation (ChIP) assays utilizing control or ethanol treated mouse liver tissues revealed that binding of ERRγ to the FGF23 promoter is significantly increased by alcohol treatment (Fig. 3A). Moreover, WT mice were infected with mouse FGF23 promoter WT-luciferase (Ad-FGF23 WT-luc) or ERRE mutant-luciferase (Ad-FGF23 mut-luc) constructs via tail vein injection, and treated with vehicle or ethanol intragastically for 12 h after four days of infection. *In vivo* imaging of the mouse livers showed that ethanol treatment led to an elevation in WT FGF23 promoter activity, as indicated by the increased signal. Conversely, mice injected with ERRE mutant promoter displayed a lack of response to ethanol treatment (Fig. 3B). These results indicate that ERRγ directly binds to the ERRE region of the FGF23 gene promoter to upregulate mRNA expression of FGF23. We also used hepatocyte-specific ERRγ knock-out mice (ERRγ-LKO) to further confirm the role of ERRγ in alcohol-induced upregulation of FGF23 expression *in vivo*. WT and ERRγ-LKO mice were exposed to vehicle or alcohol, then ERRγ and FGF23 mRNA and protein expression, as well as FGF23 plasma levels were measured. In agreement with the *in vitro* results, alcohol-induced upregulation of FGF23 mRNA and protein expression is significantly blunted in ERRγ-LKO mice (Fig. 3C–F). Moreover, considerably increased plasma iFGF23 levels in alcohol treated WT mice were not observed in alcohol fed ERRγ-LKO mice (Fig. 3G). We also assessed the mRNA expression of another alcohol-inducible ERRγ target gene within the FGF family, FGF21. Comparable to FGF23, alcohol treatment stimulated hepatic FGF21 mRNA expression in WT mice. However, ERRγ-LKO mice did not exhibit a response to alcohol treatment, leading to a lack of increase in FGF21 mRNA expression (Fig. S4). Taken together, these results confirm that ERRγ expression is mandatory to induce hepatic FGF23 expression in alcohol treated mice.

### 3.4 An inverse agonist of ERRγ reduces FGF23 gene expression and improves chronic alcohol-induced liver injury

**Fig. 4:**
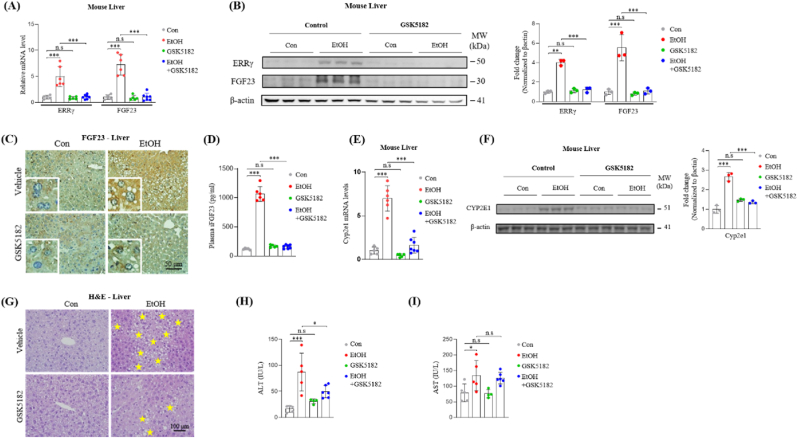
GSK5182 reduces hepatic FGF23 expression and inhibits chronic alcohol induced liver injury. (A–G) WT mice were treated with ethanol in presence or absence of GSK5182, and sacrificed for analysis (Con *n* = 5, EtOH *n* = 6, GSK5182 *n* = 5, EtOH-GSK5182 *n* = 9). (A) Quantitative PCR analysis of ERRγ and FGF23 mRNA levels in total RNA isolated from liver tissues. (B) Western blot analysis and quantification of ERRγ and FGF23 protein levels in liver tissues. (C) Representative images of FGF23 immunohistochemical analysis in liver sections. (D) Plasma iFGF23 level was measured by ELISA. (E) Quantitative PCR analysis of CYP2E1 mRNA and (F) Western blot analysis and quantification of CYP2E1 protein level from the mice livers. (G) Representative images of hematoxylin and eosin (H&E) staining in liver sections of the mice. Asterisk indicates vacuolar degeneration of hepatocytes. (H and I) Plasma levels of (H) alanine aminotransferase (ALT) and (I) aspartate aminotransferase (AST) in mice. The data were expressed as the mean ± SEM and analyzed by ordinary one-way ANOVA with Tukey's multiple comparisons test. *p < 0.05; **p < 0.01; ***p < 0.001; not significant (*n.s*).

As ERRγ was found to the key regulator of alcohol induced FGF23 gene expression, we intended to investigate the effect of pharmacological inhibition of ERRγ using an inverse agonist, GSK5182. WT mice were exposed to alcohol for 4 weeks in presence or absence of GSK5182, and then mRNA and protein expressions as well as liver injury markers were assessed. Alcohol-induced up-regulation of FGF23 mRNA and protein expression along with elevated plasma FGF23 levels were significantly reduced by GSK5182 treatment (Fig. 4A–D). As reported in our previous study [26] and our observation in the current study (Fig. 1B), CYP2E1 expression was found to be increased by alcohol treatment, which is abrogated by GSK5182 treatment (Fig. 4 E and F). Finally, H&E staining of liver sections and plasma levels of liver injury markers were assessed in alcohol exposed mice. GSK5182 treatment substantially reduced alcohol-induced macrovesicular steatosis with ballooned hepatocytes, as shown by H&E staining and significant reduction of plasma liver injury markers (Fig. 4G–I). These results imply that ERRγ-specific inverse agonist significantly reduces hepatic FGF23 production and inhibits alcoholic liver injury.

### 3.5 Hepatic FGF23 deficiency inhibits alcohol-induced CYP2E1 induction and liver injury

**Fig. 5:**
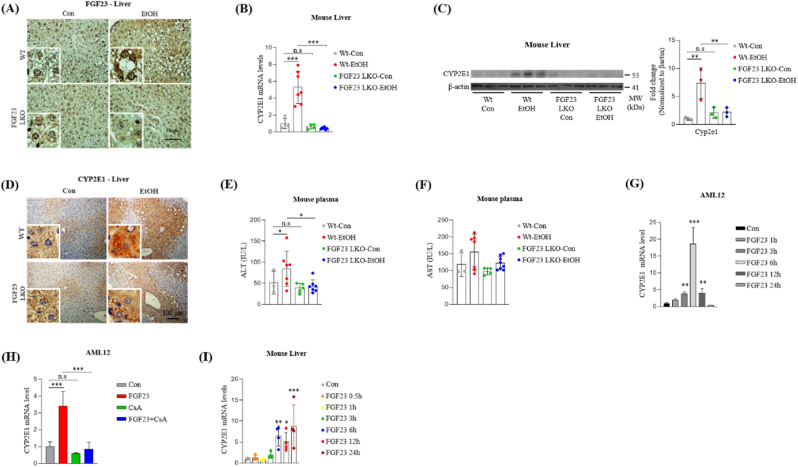
Hepatocyte specific FGF23 deficiency inhibits alcohol mediated CYP2E1 expression and liver injury. (A–F) WT and hepatocyte specific FGF23 knock-out (FGF23-LKO) mice were treated with vehicle or ethanol and sacrificed for analysis (Wt-Con n = 4, Wt-EtOH n = 7, FGF23-LKO-Con n = 4, FGF23-LKO-EtOH n = 8). (A) Representative images of FGF23 immunohistochemical analysis in liver sections. (B) Quantitative PCR analysis of CYP2E1 mRNA level in total RNA isolated from mouse liver. (C) Western blot analysis and quantification of CYP2E1 protein expression in liver tissues. (D) Representative images of CYP2E1 immunohistochemical analysis in liver sections. (E and F) Plasma levels of (E) alanine aminotransferase (ALT) and (F) aspartate aminotransferase (AST) in mice. (G) Quantitative PCR analysis of CYP2E1 mRNA level in total RNA isolated from AML12 cells treated with human recombinant FGF23 (50 ng/ml) in time dependent manner. (H) Quantitative PCR analysis of CYP2E1 mRNA level in total RNA isolated from AML12 cells treated with human recombinant FGF23 (50 ng/ml) for 6 h in presence or absence of cyclosporine A (1 μg/ml). (I) Quantitative PCR analysis of CYP2E1 mRNA level in total RNA isolated from the liver tissues of WT mice treated with human recombinant FGF23 (40 μg/kg in PBS) in time dependent manner. All *in vitro* experiments were conducted in triplicates as independent biological replicates. The data were expressed as the mean ± SEM and analyzed using two-tailed Student's *t*-test (I and K) or ordinary one-way ANOVA with Tukey's multiple comparisons test. *p < 0.05; **p < 0.01; ***p < 0.001; not significant (n.s).

CYP2E1 is a key enzyme contributing to oxidative liver injury through generation of ROS in response to alcohol exposure. It will be informative to investigate the possible role of alcohol-induced FGF23 in regulating CYP2E1 expression in the liver. For this purpose, we generated hepatocyte-specific FGF23 knock-out mice (FGF23-LKO) and confirmed that ethanol induced up-regulation of hepatic FGF23 gene expression and elevated plasma FGF23 levels were not detected in FGF23-LKO mice (Fig. S5 A-C and Fig. 5A). Moreover, hepatocyte specific deletion of FGF23 expression significantly inhibits alcohol mediated up-regulation of CYP2E1 mRNA and protein expression, as evident from RT-qPCR, Western blot and immunohistochemical analyses of mice liver (Fig. 5 B-D and Fig. S5 D). Additionally, level of the liver injury marker ALT is significantly reduced in the plasma of FGF23-LKO mice compared to WT mice treated with alcohol, whereas AST levels showed a tendency of decrease (Fig. 5E and F). For *in vitro* studies, we used cyclosporine A (CsA), a calcineurin inhibitor that blocks FGF23-calcinurin/NFAT signal transduction, thereby inhibiting FGF23 downstream signaling, to unravel the possible role of FGF23 in CYP2E1 gene regulation. Firstly, we evaluated CYP2E1 expression level in recombinant FGF23 treated AML12 cells, and identified that FGF23 significantly increases CYP2E1 mRNA expression in a time dependent manner (Fig. 5G), and this effect is blocked in cells that were pre-treated by CsA (Fig. 5H). To confirm this FGF23 effect on CYP2E1 expression *in vivo*, WT mice were intravenously injected with recombinant FGF23. In accordance with *in vitro* results, hepatic CYP2E1 mRNA expression was found to be increased in a time dependent manner (Fig. 5I). These results revealed that hepatic FGF23 is required to induce CYP2E1 expression in chronic alcohol diet-fed mice.

### 3.6 Hepatocyte specific FGF23 deficiency protected mice against chronic alcohol-induced liver injury through inhibiting oxidative stress and apoptosis

**Fig. 6:**
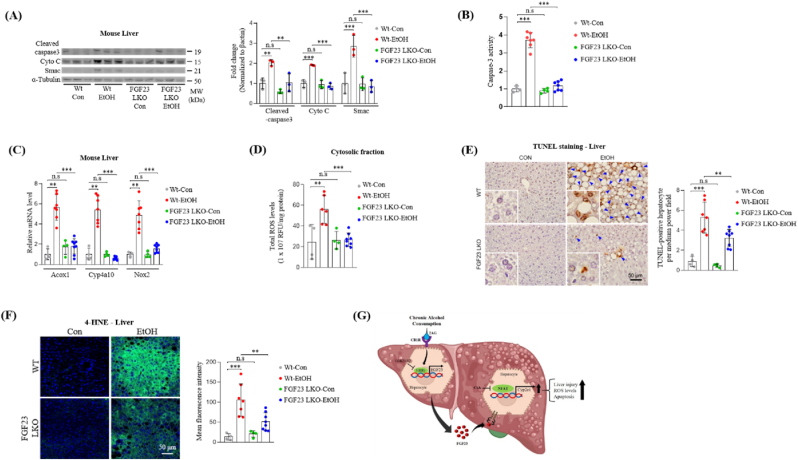
Hepatocyte specific FGF23 deficiency protects the mice against chronic alcohol-induced oxidative injury and apoptosis. (A–F) WT and hepatocyte specific FGF23 knock-out (FGF23-LKO) mice were treated with vehicle or ethanol and sacrificed for analysis (Wt-Con n = 4, Wt-EtOH n = 7, FGF23-LKO-Con n = 4, FGF23-LKO-EtOH n = 8). (A) Western blot analysis and quantification of cleaved caspase 3, cytochrome C and Smac protein levels in cytosolic fraction of the liver tissues. (B) Calorimetric quantification of caspase-3 activity is represented as fold change with respect to WT-control group (Wt-Con n = 4, Wt-EtOH n = 6, FGF23-LKO-Con n = 4, FGF23-LKO-EtOH n = 8). (C) Quantitative PCR analysis of ROS-generating enzymes mRNA levels from the total RNA of mouse livers. (D) Measurement of total ROS production in the cytosolic fraction of the mouse liver tissues. (E) Representative images of TUNEL assay and quantification of TUNEL positive hepatocytes in liver sections of ethanol treated WT and FGF23-LKO mice. Arrowheads indicate hepatocytes that are positive for TUNEL staining. (F) Representative immunofluorescence images of 4-hydroxynonenal (4-HNE) staining and quantification of mean fluorescent intensity in liver sections of ethanol treated WT and FGF23-LKO mice. (G) Schematic model of alcohol-CB1R-ERRγ-FGF23-CYP2E1 signal transduction axis-mediated oxidative stress and alcoholic liver injury. The data were expressed as mean ± SEM and analyzed using ordinary one-way ANOVA with Tukey's multiple comparisons test. *p < 0.05; **p < 0.01; ***p < 0.001; not significant (n.s).

To examine the mechanism of FGF23-mediated alcoholic liver damage, we treated WT and FGF23-LKO mice with alcohol and assessed Smac and cytochrome C-mediated apoptotic pathways. Smac is a mitochondrial protein known to potentiate caspase induced cell apoptosis through cytochrome C. In response to alcohol exposure, WT mice showed upregulated levels of smac, cytochrome *c*, and cleaved caspae-3 protein. However, this effect was blunted in mice with hepatocyte-specific depletion of FGF23 (Fig. 6A). We also observed the absence of alcohol-mediated upregulation of caspase-3 activity in the liver tissues of FGF23-LKO mice compared to WT mice (Fig. 6B). Alcohol-induced hepatic upregulation of mRNA of ROS-generating enzymes, acyl-Coenzyme A oxidase 1 (Acox1), cytochrome P450 4A10 (CYP4A10) and NADPH oxidase 2 (NOX2) are significantly decreased upon depletion of FGF23 (Fig. 6C). In harmony with the gene expression levels of ROS-generating enzymes, the total ROS levels in the cytosolic fraction of alcohol treated mouse liver cells was significantly lower in FGF23-LKO compared to WT mice (Fig. 6D). Assessing hepatocyte cell death by TUNEL assay revealed that loss of hepatocyte FGF23 expression significantly inhibited hepatocyte apoptosis in chronic alcohol fed mice (Fig. 6E). Moreover, immunofluorescence staining of 4-HNE in liver sections of alcohol fed mice revealed that hepatic FGF23 deficiency protected the liver from alcohol-induced oxidative damage (Fig. 6F). We assessed the blood alcohol concentration in both WT and FGF23-LKO mice and found no significant difference between them (Fig. S6A). This finding eliminates the possibility that variation in alcohol intake could account for the observed protective effect in FGF23-LKO mice against alcohol-induced liver injury. Moreover, we also measured hepatic FGF21 mRNA expression and secretory FGF21 levels in vehicle or ethanol treated WT and FGF23-LKO mice. No significant difference was detected between WT and FGF23-LKO mice in terms of both hepatic FGF21 mRNA and plasma FGF21 levels (Figs. S6B and S6C). Taken together, these results indicate that loss of hepatic FGF23 expression protects the liver from chronic alcohol damage through inhibiting ROS generation, oxidative damage and apoptosis.

## 4 Discussion

Our study revealed that activated CB1R-induced ERRγ regulates hepatic FGF23 production in hepatocytes, which in turn regulates CYP2E1 gene expression and facilitates liver injury upon chronic alcohol consumption. Chronic alcohol exposure induces ERRγ and FGF23 gene expression specifically in the liver, and ERRγ was identified as transcriptional regulator of alcohol-induced hepatic FGF23 expression. Hepatocyte specific genetic depletion of CB1R or ERRγ significantly reduced alcohol-induced hepatic FGF23 production and pharmacological inhibition of ERRγ transactivation is sufficient to reduce hepatic FGF23 expression, plasma FGF23 levels, hepatic CYP2E1 expression and liver injury in chronic alcohol-fed mice. Finally, hepatocyte-specific loss of FGF23 inhibited CYP2E1 expression and prevented chronic alcohol-induced liver injury through inhibiting hepatic ROS generation and apoptosis in mice.

Bone is the primary source of FGF23 under biological homeostasis, however in pathological conditions FGF23 is also produced from multiple non-osseous tissues including heart, thymus, spleen and liver [34,48,49]. Hepatic production of FGF23 was previously reported in autosomal dominant polycystic kidney disease, childhood biliary atresia and end-stage liver disease patients [32,33,50]. In mice, activation of Janus kinase-signal transducer and activator of transcription 3 pathway in hepatic inflammation leads to FGF23 production from the liver [51]. Moreover, the mouse models of FA-AKI and CCl_4_-ALI also reported hepatic FGF23 production [24,25]. We previously showed FGF23 production from the liver through ERRγ in FA-AKI mice, and that liver specific ERRγ knock-out or an inverse agonist of ERRγ significantly, but not completely, reduced circulatory FGF23 levels. In FA-AKI mice, besides the liver other major organs like heart, spleen and bone also produce FGF23 and account for remaining FGF23 levels in the circulation after obstructing hepatic FGF23 production [24]. Circulatory FGF23 levels are reported to be increased among alcoholics [36,37], however the source of FGF23, its mechanism of regulation and role in ALD is unknown. Interestingly, in our current study, we showed that FGF23 is mainly expressed in hepatocytes and its expression is induced in livers of alcoholic hepatitis and alcoholic cirrhosis patients (Fig. 1A–C). Importantly, the data in mice and cell culture robustly display FGF23 as a critical factor upstream of CYP2E1 expression, the enzyme that is metabolizing excess amounts of alcohol in settings, where alcohol dehydrogenase is not sufficient. The inhibition of alcohol-induced upregulation of hepatic CYP2E1 expression in FGF23-LKO mice (Fig. 5B–D) underscores the role of FGF23 in regulating CYP2E1 expression in response to alcohol. FGF23 upregulates hepatic CYP2E1 expression potentially through FGF23-PLCγ/Calcineurin-nuclear factor of activated T cells (NFAT) signaling pathway. Previous studies have demonstrated that FGF23 regulates target gene expression by activating the PLCγ/Calcineurin pathway in hepatocytes and cardiac myocytes [[52], [53], [54], [55]]. In this study, we employed a calcineurin inhibitor, cyclosporine A (CysA), to illustrate the involvement of the PLCγ/Calcineurin pathway in FGF23-mediated upregulation of hepatic CYP2E1 expression (Fig. 5H). Our previous study indicated that ERRγ transcriptionally regulates hepatic CYP2E1 expression in response to alcohol-activated CB1R signaling [26]. In this study, we revealed ERRγ-induced FGF23 as a novel pathway in the regulation of alcohol-induced hepatic CYP2E1 expression. Consequently, GSK5182, an inverse agonist of ERRγ, mitigates alcohol-induced hepatic CYP2E1 expression by inhibiting ERRγ transactivation, thereby disturbing the ERRγ-FGF23-CYP2E1 signaling axis (Fig. 4E and F). We also proved that genetic abrogation of hepatic ERRγ expression or pharmacological inhibition of ERRγ transactivation totally inhibit alcohol-induced FGF23 production, and plasma FGF23 levels of ERRγ-LKO or GSK5182 treated ALD mice are equivalent to non-alcohol treated control mice (Fig. 3, Fig. 4). Therefore, unlike in the kidney damage condition, liver is the only source of circulatory FGF23 in chronic alcohol-induced liver injury through hepatocyte specific activation of the endocannabinoid-CB1R-ERRγ signal transduction axis.

FGF21, along with FGF19 (known as FGF15 in mammals) and FGF23, belongs to the FGF19 subfamily, which represents the endocrine members of the broader FGF family. FGF21 is a stress inducible peptide hormone predominantly produced from hepatocytes in response to metabolic signals. It plays an important role in various physiological and pathological processes like lipolysis, gluconeogenesis, thermogenesis, insulin sensitivity, energy homeostasis, inflammation, oxidative, and/or endoplasmic reticulum stress in major organs like liver, brain, heart, kidney, adipose tissue, skeletal muscle, and pancreas [56,57]. Unlike other FGF family members, FGF19 subfamily members lack the heparin binding domain and hence are liberally released into the circulation. We noticed a number of similarities between FGF21 and FGF23 that is (i) liver injury increases both hepatic FGF21 and FGF23 production [25,49,58], (ii) hepatocytes are the source of FGF21 and FGF23 in liver injury [25,59], however a previous report also showed FGF23 production from liver residing macrophages [49], (iii) klotho co-receptors are mandatory for proper signal transduction, α-klotho for FGF23 and β-klotho for FGF21 [60] and (iv) most interestingly, our previous reports showed that an orphan nuclear receptor ERRγ transcriptionally regulates the gene expression of both FGF21 and FGF23 in the liver [24,25,27]. On top of these resemblances, FGF21 and FGF23 plays an opposing role in alcohol-induced liver injury. It was reported that FGF21 is hepatoprotective in alcoholic liver injury [[61], [62], [63]], whereas here we describe a pathological role of FGF23 in chronic alcoholic liver injury through increasing hepatic CYP2E1 production, enhancing ROS generation and inducing hepatocyte apoptosis (Fig. 5, Fig. 6). We established that there is no significant difference in both hepatic FGF21 mRNA expression and secretory FGF21 levels between WT and FGF23-LKO mice in response to alcohol treatment (Figs. S6B and S6C). This finding rules out the possibility that a hepato-protective effect of FGF21 could account for the observed protection against alcoholic liver damage in FGF23-LKO mice. It is worth to note that in different disease models, pharmacological inhibition of ERRγ transactivation significantly reduced both FGF21 and FGF23 production in the mice liver [24,25,27]. The upstream metabolic signals through specific membrane receptors determines the downstream target genes of ERRγ [23]. We can consider ERRγ as a double-edged sword, which can regulate the gene expression of hepato-protective FGF21 as well as hepato-damaging FGF23 in hepatocytes. In-depth examination about the determining factors of ERRγ target gene specificity will shed light on the way of precisely modulating the target genes expression to treat various liver diseases.

In conclusion, we report that activated CB1 receptor induced ERRγ expression leads to hepatic FGF23 expression, which in turn up-regulates CYP2E1 expression and thereby exacerbates chronic alcohol-induced liver injury in mice. Hepatocyte-specific genetic depletion of CB1R abolished alcohol dependent up-regulation of both ERRγ and FGF23 hepatic expression and loss of hepatic ERRγ significantly inhibited FGF23 expression induced by chronic alcohol exposure. Moreover, ERRγ-specific inverse agonist prevented alcohol mediated induction of FGF23 and CYP2E1 mRNA, FGF23 protein and plasma FGF23 levels. Finally, hepatocyte-specific loss of FGF23 prevented chronic alcoholic liver injury through reducing ROS production and inhibiting apoptosis of hepatocytes. Therefore, we suggest that CB1R-ERRγ-FGF23 signal transduction axis plays a pathological role in chronic alcoholic liver injury through regulating CYP2E1 gene expression (Fig. 6G), and FGF23 may represent a potential therapeutic target to treat alcoholic liver disease.

## Funding

This work was supported by the National Research Foundation (NRF) basic science research program Korean government (MSIT), Republic of Korea (YS Jung, NRF-2020R1A6A3A01096145); (D-K Kim, RS-2023-00219517); (Y–H Kim NRF-2019R1C1C1005319); (C–H Lee NRF-2023R1A2C3005244); and (H–S Choi, NRF-2021R1A2C3004923 and RS-2023-00219517) and the Korea Research Institute of Bioscience and Biotechnology (KRIBB) Research Initiative Program (KGM5212322). Korean Health Technology R&D project through the Korea Health Industry Development Institute (KHIDI), funded by the Ministry of Health & Welfare, Republic of Korea (HI16C1501). The Federal Ministry of Education and Research-Liver Systems Medicine Program LiSyM-Cancer, Grant PTJ-031L0257A, and by the Stiftung für Biomedizinische Alkoholforschung.

## CRediT authorship contribution statement

**Yoon Seok Jung:** Writing – review & editing, Methodology, Investigation, Funding acquisition, Data curation, Conceptualization. **Kamalakannan Radhakrishnan:** Writing – review & editing, Writing – original draft, Investigation, Formal analysis, Conceptualization. **Seddik Hammad:** Investigation, Data curation. **Sebastian Müller:** Investigation, Data curation. **Johannes Müller:** Investigation, Data curation. **Jung-Ran Noh:** Investigation, Data curation. **Jina kim:** Investigation, Data curation. **In-Kyu Lee:** Formal analysis, Data curation. **Sung Jin Cho:** Investigation, Data curation. **Don-Kyu Kim:** Writing – review & editing, Writing – original draft, Formal analysis. **Yong-Hoon Kim:** Writing – review & editing, Investigation, Funding acquisition, Data curation, Conceptualization. **Chul-Ho Lee:** Writing – review & editing, Supervision, Funding acquisition, Formal analysis, Conceptualization. **Steven Dooley:** Writing – review & editing, Supervision, Funding acquisition, Formal analysis, Data curation, Conceptualization. **Hueng-Sik Choi:** Writing – review & editing, Writing – original draft, Supervision, Funding acquisition, Conceptualization.

## Declaration of competing interest

The authors declare that they have no known competing financial interests or personal relationships that could have appeared to influence the work reported in this paper.

## Data Availability

Data will be made available on request.
