# Supplementary material for: ERRγ-inducible FGF23 promotes alcoholic liver injury through enhancing CYP2E1 mediated hepatic oxidative stress
Source: Redox Biol. 2024 Mar 5;71:103107. doi: 10.1016/j.redox.2024.103107 (PMC10950689; doi:10.1016/j.redox.2024.103107)
Supplement: Multimedia component 1 [file mmc1.docx]

**SUPPLEMENTARY INFORMATION**

**ERRγ-inducible hepatokine FGF23 promotes alcoholic liver injury through enhancing CYP2E1 mediated hepatic oxidative stress**

Yoon Seok Jung ^a, 1^, Kamalakannan Radhakrishnan ^a, 1^, Seddik Hammad ^b, c^, Sebastian Müller ^d^, Johannes Müller ^d^, Jung-Ran Noh ^e^, Jina kim ^f^, In-Kyu Lee ^g, h^, Sung Jin Cho ^i^, Don-Kyu Kim^j^, Yong-Hoon Kim ^e, k *^, Chul-Ho Lee ^e, k *^, Steven Dooley ^b *^, Hueng-Sik Choi ^a*^

**Corresponding author:** Hueng-Sik Choi

**Email:** hsc@chonnam.ac.kr

**This supplementary file includes:**

1. Supplementary Methods
2. Supplementary Figures S1 to S6
3. Supplementary Table1

**1. SUPPLEMENTARY METHODS**

- 1. *Hepatic Zonation of FGF23 expression in healthy human liver and chronic alcohol-fed mice liver*

Data regarding the basal expression levels of FGF23 in different zones of human liver tissues were extracted from publicly available Gene Expression Omnibus (GEO) database, specifically from the dataset GSE105127. In mice, WT mice were treated with vehicle or ethanol and sacrificed for analysis. The protein expression pattern of FGF23 was analyzed in three main areas of hepatic lobule: periportal zone, intermediate zone, and pericentral zone through immunohistochemistry of FGF23. Representative images were presented.

- 1. *Measuring plasma alcohol concentration*

Ethanol assay kit (Abcam, catalog # ab65343, Cambridge, USA) was utilized to measure alcohol concentration from plasma of mice according to the manufacturer’s protocol. In brief, plasma samples were added in duplicates to the wells of a 96-well plate, and the volume was adjusted to 50 µL/well using assay buffer. Subsequently, 50 µL of the reaction mix was added to each well. Only ethanol assay buffer is used in the background control wells and 50 µL/well standard dilutions were used for standard curve preparation. The plate was incubated at 37°C for 60 minutes, shielded from light. Absorbance at 570 nm was measured for all wells using a microplate reader.

- 1. *Measuring plasma FGF21 levels*

Concentration of circulatory FGF21 levels were measured from mouse plasma using a mouse FGF21 ELISA kit (Abcam, catalog # ab212160, Minneapolis, MN, USA) according to the manufacturer’s protocol.

1. **SUPPLEMENTARY FIGURES**


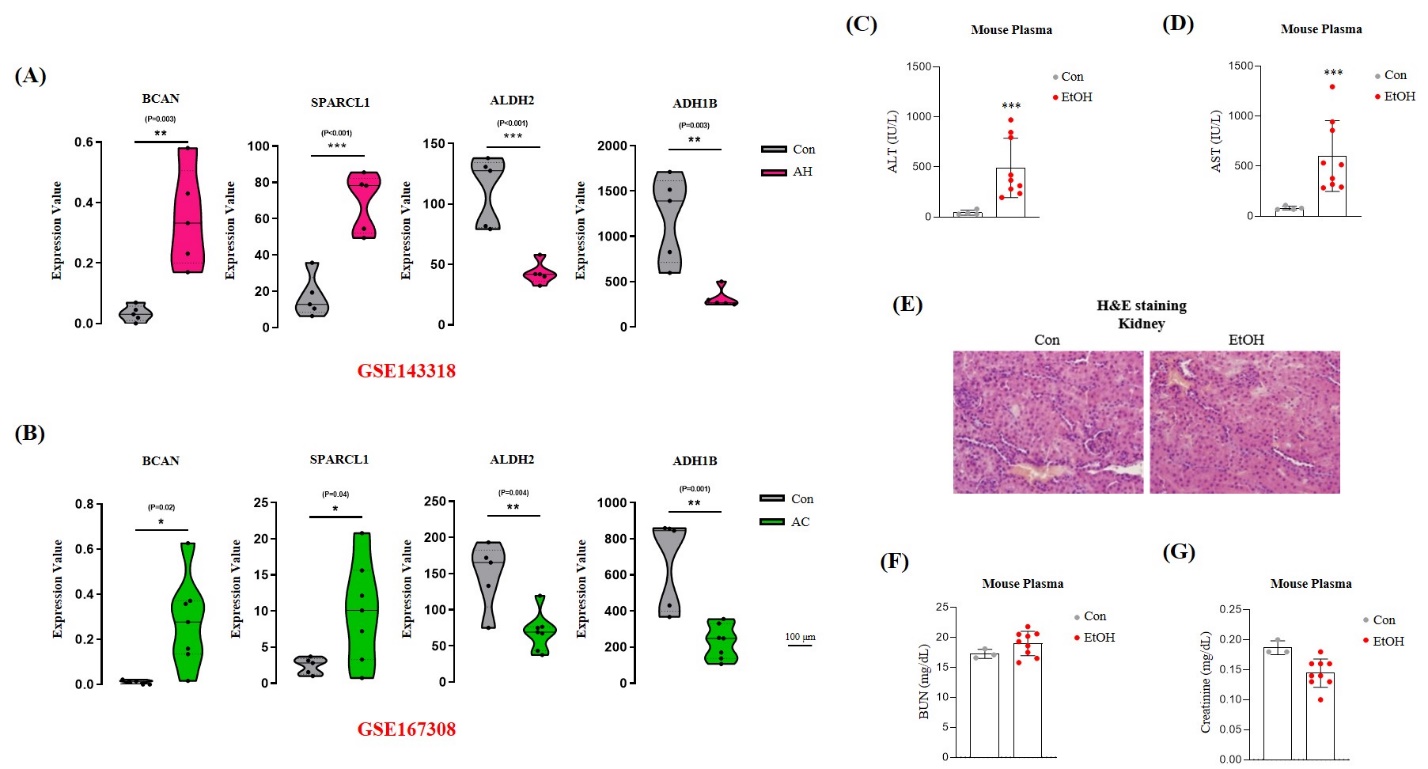


Fig. S1. Expression levels of validation genes for Fig. 1A & B; and analysis of liver and kidney injury markers of alcoholic liver injury mice. (A, B) Hepatic levels of BCAN, SPARCL1, ALDH2 and ADH1B expression in alcoholic hepatitis and cirrhosis patients compared to healthy controls using two datasets obtained from the Gene Expression Omnibus (GEO) database (GSE143318-alcoholic hepatitis; GSE167308-alcoholic cirrhosis). (C-G) WT mice were treated with ethanol and sacrificed after 4 weeks (con *n*=5, EtOH *n*=9). (C, D) Plasma levels of (C) alanine aminotransferase (ALT) and (D) aspartate aminotransferase (AST). (E) Representative images of hematoxylin and eosin (H&E) staining in liver sections. (F, G) Plasma levels of (F) blood urea nitrogen (BUN) and (G) creatinine in control and ethanol treated mice. The data were expressed as the mean ± SEM and analyzed using two-tailed Student’s *t* test. **p* < 0.05; ***p* < 0.01; ****p* < 0.001.

**
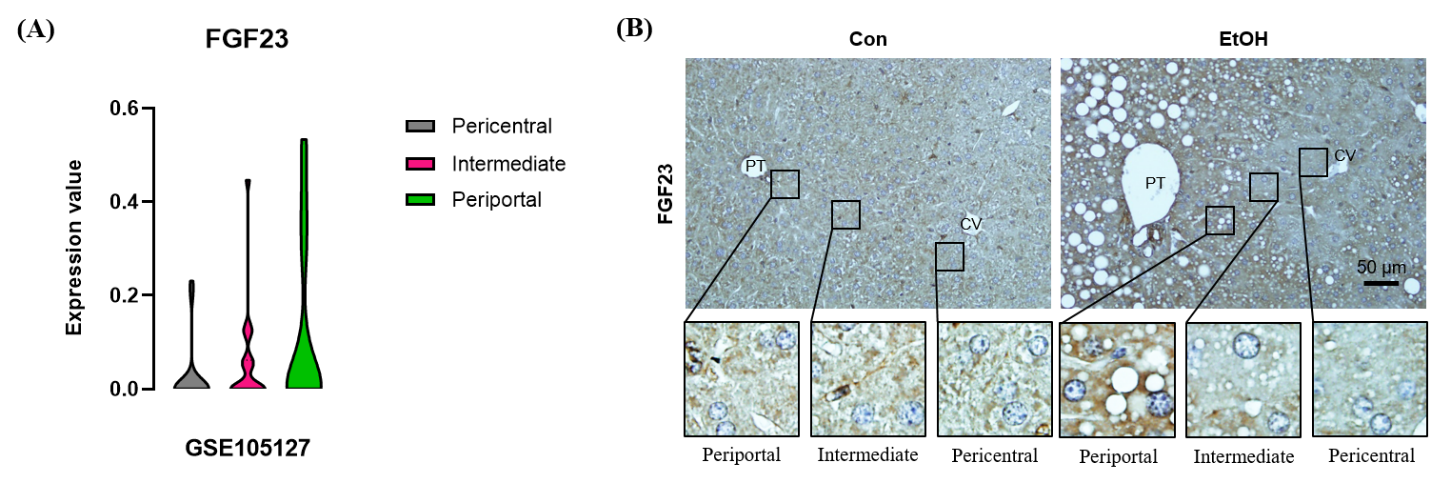
**

Fig. S2. Expression levels of FGF23 in different zones of the human and mouse livers. (A) Basal expression levels of FGF23 in pericentral, intermediate, and peripheral zones of human liver. Data extracted from publicly available Gene Expression Omnibus (GEO) database with the dataset number GSE105127. (B) Representative images of FGF23 immunohistochemistry analysis of liver sections from control and ethanol fed WT mice. The protein expression pattern of FGF23 was analyzed in pericentral, intermediate, and peripheral zones of mice livers. PT, portal triad; CV, central vein.

**
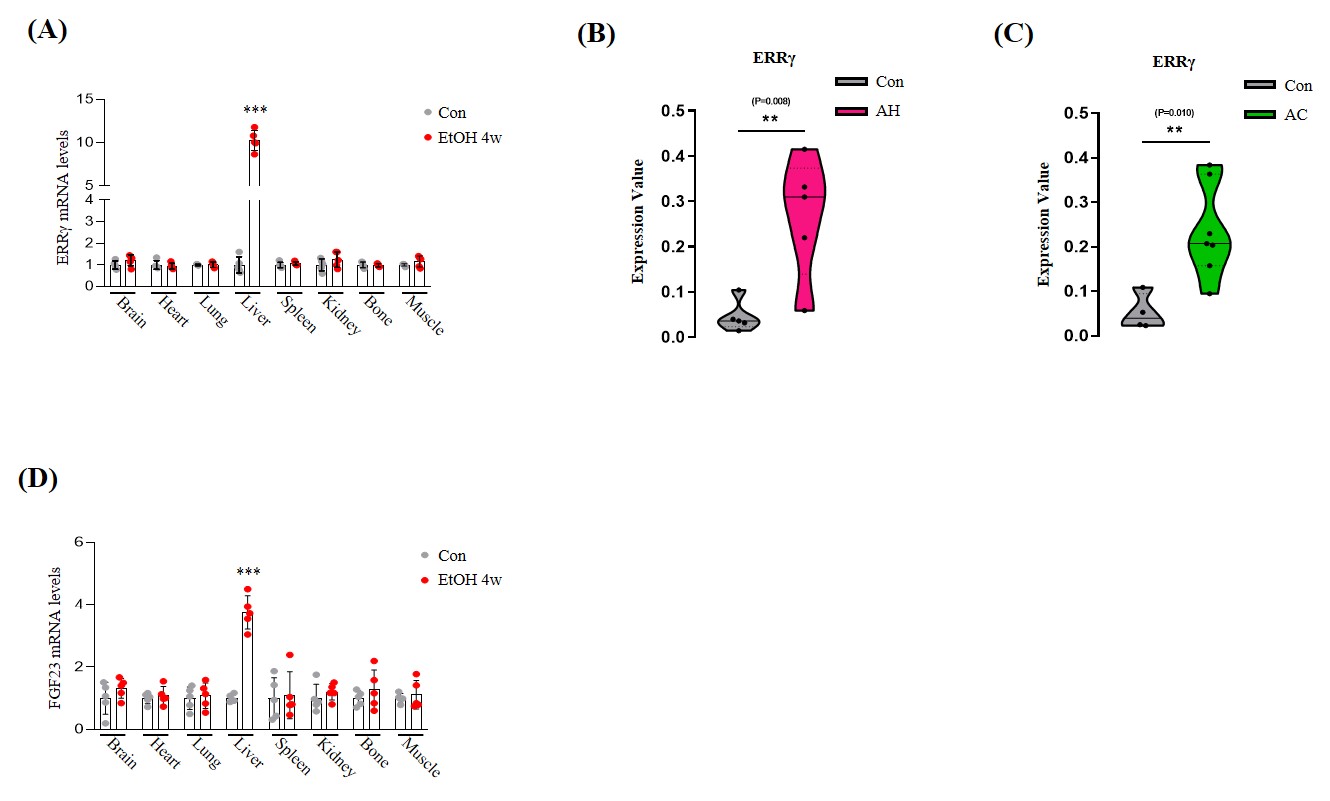
**

Fig. S3. ERRγ expression in ALD patients and mice. (A) Quantitative PCR analysis of ERRγ mRNA level in total RNA isolated from control or ethanol ingested mouse livers (*n*=5 per group). (B, C) Hepatic levels of ERRγ expression in alcoholic hepatitis and cirrhosis patients compared to healthy controls using two datasets obtained from the Gene Expression Omnibus (GEO) database (GSE143318-alcoholic hepatitis; GSE167308-alcoholic cirrhosis). (D) Quantitative PCR analysis of FGF23 mRNA level in total RNA isolated from control or ethanol ingested mouse livers (*n*=5 per group). The Data were expressed as the mean ± SEM and analyzed using two-tailed Student’s *t* test. ***p* < 0.01; ****p* < 0.001.


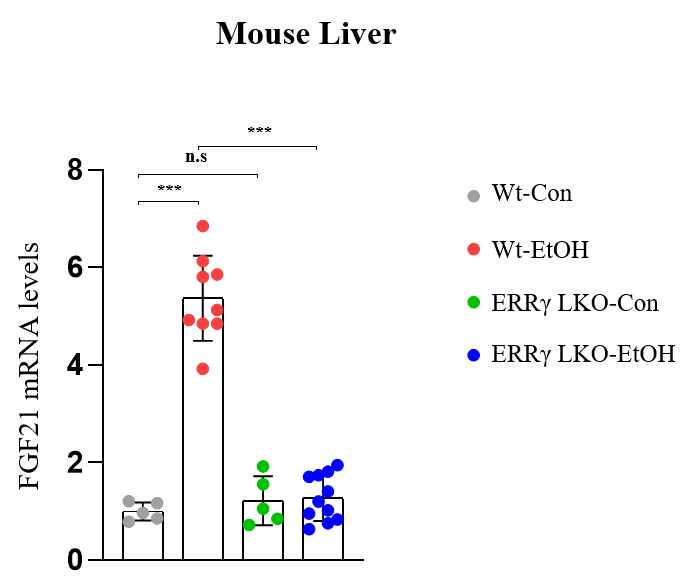


Fig. S4. FGF21 mRNA expression level in WT and ERRg-LKO mice fed with ethanol. Quantitative qPCR analysis of FGF21 mRNA level in total RNA isolated from control and ethanol fed WT and ERRg-LKO mice (Wt-Con n=5, Wt-EtOH n=9, ERRγ-LKO-Con n=5, ERRγ-LKO-EtOH n=11). The Data were expressed as the mean ± SEM and analyzed using two-tailed Student’s *t* test. ****p* < 0.001; not significant (n.s).

**
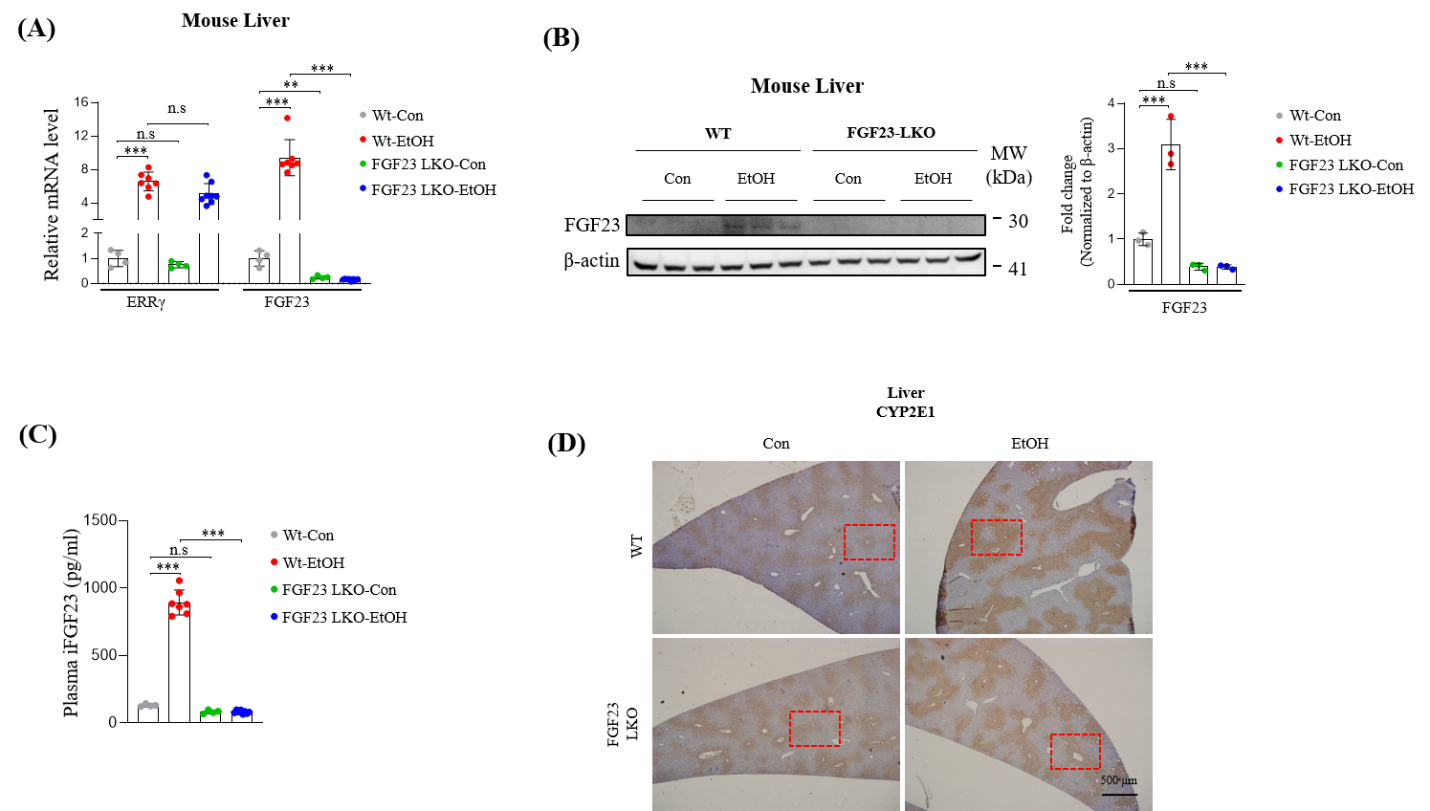
**

Fig. S5. (A-D) WT and hepatocyte specific FGF23 knock-out (FGF23-LKO) mice were treated with vehicle or ethanol and sacrificed for analysis (Wt-Con n=4, Wt-EtOH n=7, FGF23-LKO-Con n=4, FGF23-LKO-EtOH n=8). (A) Quantitative PCR analysis of ERRγ and FGF23 mRNA levels in total RNA isolated from mouse liver. (B) Western blot analysis and quantification of FGF23 protein level in liver tissues (n=3 per groups). (C) Plasma iFGF23 levels were measured by ELISA. (D) Low-magnification light microscope images for Fig 5D. Representative images of immunohistochemistry for CYP2E1 in liver tissues of WT and FGF23-LKO mice treated with ethanol. The rectangle indicates the area of high-magnification light microscope images shown in Fig 5D.


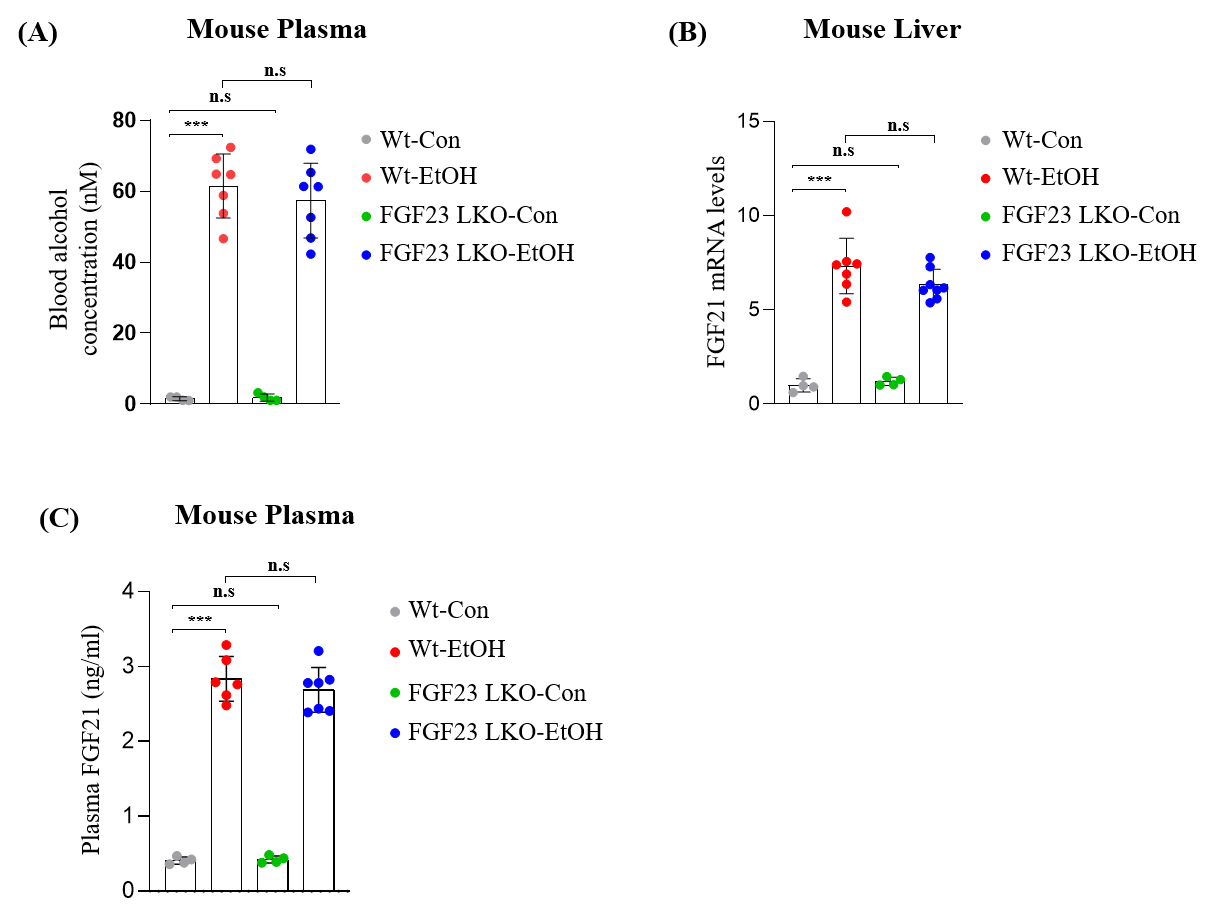


Fig. S6. Blood alcohol concentration, FGF21 mRNA expression and FGF21 secretory levels in WT and FGF23-LKO mice fed with ethanol. (A) Alcohol concentration in plasma of control and ethanol fed WT and FGF23-LKO mice (Wt-Con n=4, Wt-EtOH n=7, FGF23-LKO-Con n=4, FGF23-LKO-EtOH n=7). (B) Quantitative qPCR analysis of FGF21 mRNA level in total RNA isolated from control and ethanol fed WT and FGF23-LKO mice (Wt-Con n=4, Wt-EtOH n=7, FGF23-LKO-Con n=4, FGF23-LKO-EtOH n=8). (C) Plasma FGF21 levels were measured by ELISA from control and ethanol fed WT and FGF23-LKO mice (Wt-Con n=4, Wt-EtOH n=6, FGF23-LKO-Con n=4, FGF23-LKO-EtOH n=7). The Data were expressed as the mean ± SEM and analyzed using two-tailed Student’s *t* test. **p < 0.01; ****p* < 0.001; ns, not significant (n.s).

1. **SUPPLEMENTARY TABLE**

**Supplementary table1.** Sequences of primers used in this study

**
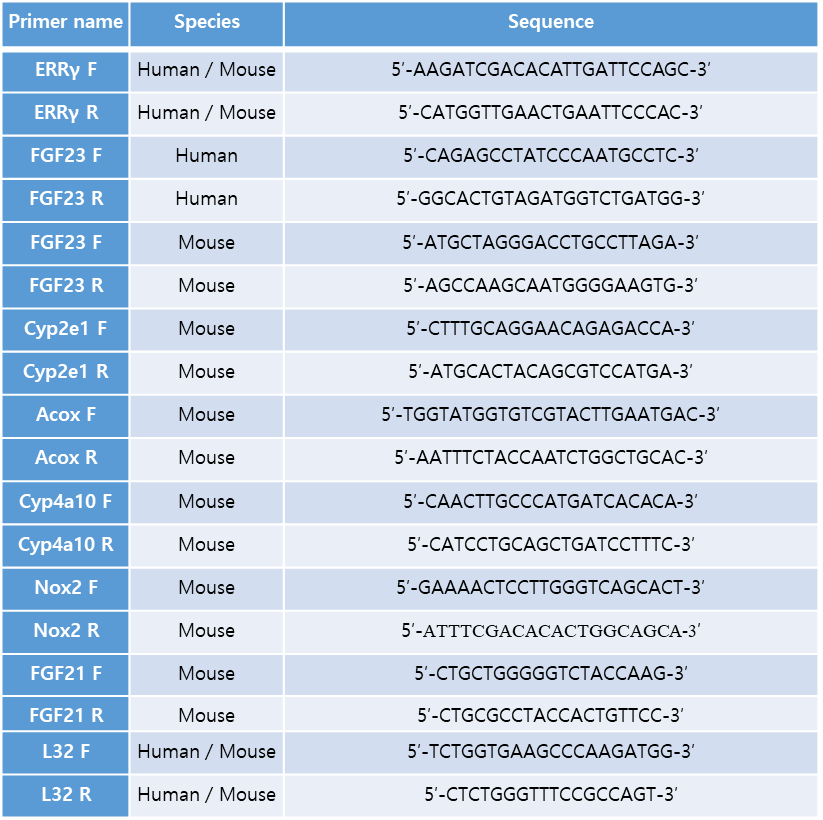
**
